# Geographic variation in proportions meeting the waist circumference criterion and having elevated hemoglobin A1c among Specific Health Checkup examinees in Japan: an ecological analysis of the 10th NDB Open Data

**DOI:** 10.1265/ehpm.26-00125

**Published:** 2026-08-18

**Authors:** Hirotaka Nakashima, Takahiro Kamihara, Takuya Omura

**Affiliations:** 1 Independent Researcher, Nagoya, Japan; 2 Department of Cardiology, Hospital, National Center for Geriatrics and Gerontology, Obu, Japan; 3 Department of Metabolic Research, Research Institute, National Center for Geriatrics and Gerontology, Obu, Japan; 4 Department of Diabetes and Endocrinology, Hospital, National Center for Geriatrics and Gerontology, Obu, Japan

**Keywords:** National Database, NDB Open Data, Specific health checkups, Hemoglobin A1c, Waist circumference, Body mass index, Ecological study, Japan

## Abstract

**Background:**

Publicly available aggregate health data can support regional surveillance of cardiometabolic risk, but analyses must address aggregation, ecological inference, and cell suppression or unavailability. Waist circumference and body mass index (BMI) capture related but distinct aspects of adiposity. We evaluated the usefulness and limitations of selected 10th National Database (NDB) Open Data indicators for prefecture-level cardiometabolic surveillance among Specific Health Checkup examinees in Japan.

**Methods:**

We conducted an ecological descriptive analysis of the 10th NDB Open Data. Data were analyzed by prefecture, sex, and 5-year age band among examinees aged 40–74 years. Primary indicators were the percentage meeting the waist circumference criterion (≥85 cm in men, ≥90 cm in women) and the percentage with hemoglobin A1c (HbA1c) ≥6.5%; BMI categories were examined as exploratory indicators. Prefecture-level percentages were internally age-standardized within sex using the age distribution of publicly displayed examinee counts pooled across prefectures for each indicator as the internal standard. Cells displayed as “-” were treated as suppressed/unavailable and not as zero values.

**Results:**

Waist circumference criterion data were complete across prefectures, sexes, and age bands. HbA1c ≥6.5% data were complete among men but included suppressed/unavailable cells among women, and BMI indicators had substantial cell suppression/unavailability. The age-standardized percentage meeting the waist circumference criterion ranged from 45.5% in Niigata to 60.6% in Okinawa among men and from 13.7% in Kyoto to 24.0% in Okinawa among women. HbA1c ≥6.5% ranged from 8.3% in Nagano to 11.3% in Miyagi among men and from 3.9% in Kanagawa to 7.8% in Saga among women. Okinawa had the highest percentage meeting the waist circumference criterion in both sexes. Waist-HbA1c correlations were positive in available-cell analyses, but estimates among women and estimates involving BMI were sensitive to cell availability and the prefectures included in the analysis.

**Conclusions:**

The percentage meeting the waist circumference criterion was the most complete and robust descriptive indicator for prefecture-level cardiometabolic surveillance using the 10th NDB Open Data. HbA1c analyses among women and BMI-based analyses require suppression-aware interpretation. Ecological correlations should be interpreted as descriptive co-patterning of aggregate surveillance indicators, not as individual-level associations or evidence of regional determinants.

**Trial registration:**

Not applicable.

**Supplementary information:**

The online version contains supplementary material available at https://doi.org/10.1265/ehpm.26-00125.

## Graphical Abstract

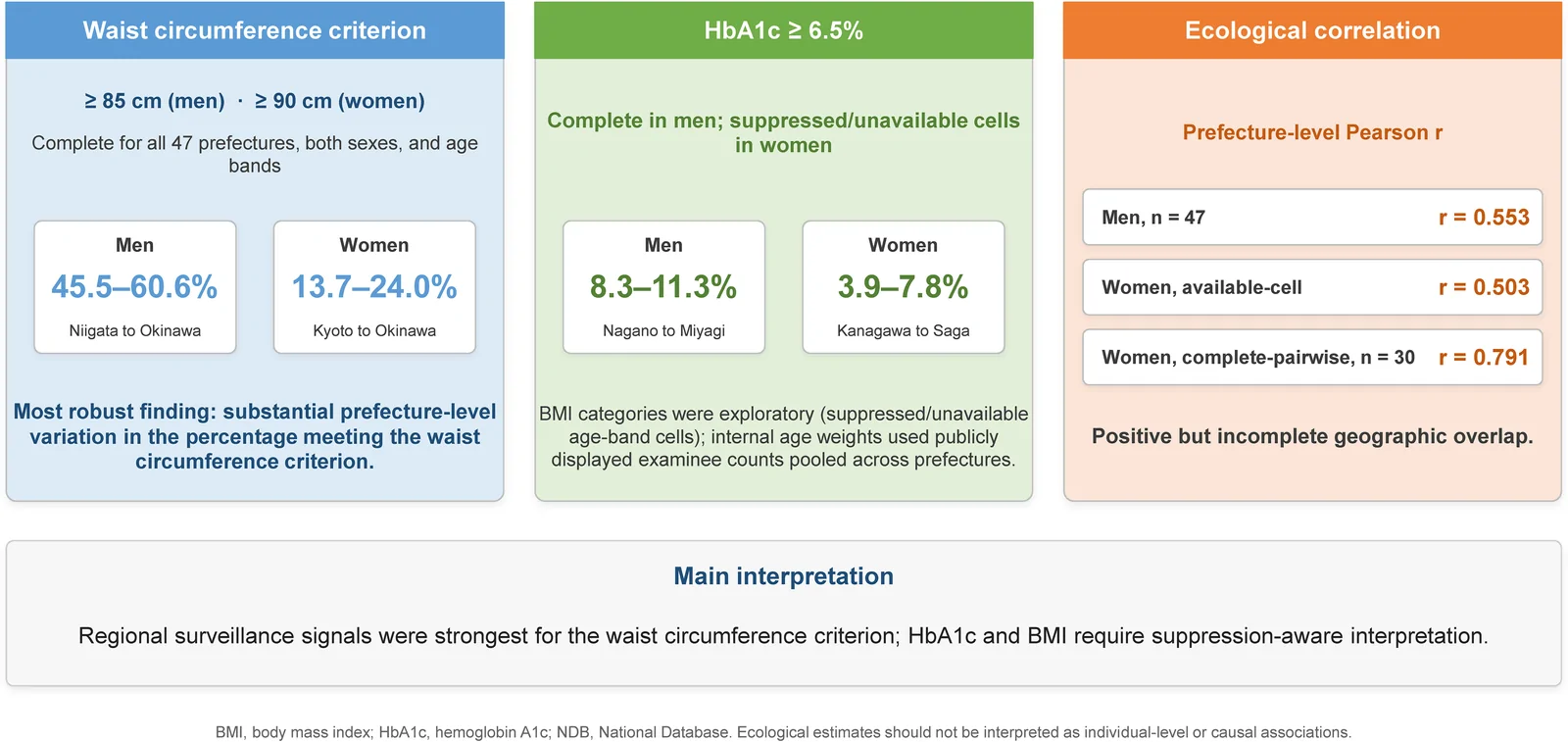

## Background

Elevated waist circumference, obesity, and dysglycemia are core cardiometabolic risk factors. Their geographic distribution may inform preventive medicine and regional surveillance even when individual-level risk-factor analyses are not available. In Japan, Specific Health Checkups and Specific Health Guidance were introduced as a nationwide program focused on metabolic syndrome prevention among adults aged 40–74 years [1, 2]. Within this program, BMI and waist circumference have different interpretive roles: BMI reflects overall body mass relative to height, whereas waist circumference is used as a practical surrogate for central adiposity and abdominal fat accumulation [3].

In the Japanese Specific Health Checkup and Specific Health Guidance framework, the waist circumference criterion has a distinctive operational role because sex-specific thresholds are incorporated into the assessment of metabolic syndrome-related risk and the selection of individuals for health guidance. Therefore, the waist circumference criterion is not only an anthropometric classification but also a programmatically important indicator aligned with a national prevention program. This role makes it particularly relevant for prefecture-level descriptive surveillance using public aggregate Specific Health Checkup data, while recognizing that waist circumference is not a direct measurement of visceral adipose tissue.

The National Database of Health Insurance Claims and Specific Health Checkups of Japan (NDB) is a national administrative database that includes health insurance claims and Specific Health Checkup data. The NDB Open Data are public aggregate tables derived from the NDB and do not include patient- or facility-level information [4, 5]. The 10th NDB Open Data include aggregated Specific Health Checkup examination data for body mass index (BMI), hemoglobin A1c (HbA1c), and waist circumference by prefecture, sex, and age band [6].

Publicly available aggregate data have important advantages for transparency and independent verification, but they also impose limitations. Analyses are ecological, cannot adjust for individual-level confounders, and may be affected by cell suppression or unavailable values. Principles for ecological studies emphasize that group-level associations cannot be assumed to represent individual-level relationships, and classic work on ecological correlations illustrates the risk of ecological fallacy [7, 8]. A careful analysis should therefore distinguish complete indicators from indicators requiring interpretation with attention to cell suppression or unavailability.

This study aimed to identify which selected 10th NDB Open Data indicators can be used most reliably for prefecture-level cardiometabolic surveillance among Specific Health Checkup examinees aged 40–74 years in Japan. Specifically, we described data completeness and cell suppression/unavailability, prefecture-level variation in the waist circumference criterion and HbA1c ≥6.5%, and ecological correlations among adiposity-related indicators and HbA1c. BMI categories were examined as exploratory indicators because of substantial cell suppression/unavailability and because BMI and waist circumference may represent different adiposity constructs at the prefecture level.

## Methods

### Study design and data source

We performed an ecological descriptive analysis of reshaped public aggregate data. The source was the 10th NDB Open Data published by the Ministry of Health, Labour and Welfare, Japan [6]. The relevant source files contained aggregated Specific Health Checkup examination data for BMI, HbA1c, and waist circumference. No additional external data were collected.

### Analytic units and indicators

The source data were organized into prefecture-sex-age-band cells covering seven 5-year age bands from 40–44 to 70–74 years; prefectures were the units of the ecological correlation analyses. Records with an unknown prefecture were retained for quality checking but excluded from prefecture-level analyses. Sex was analyzed as recorded in the NDB aggregate tables; gender-related information was not available. The primary indicators were the percentage meeting the waist circumference criterion (defined as waist circumference ≥85 cm in men and ≥90 cm in women, consistent with the Japanese Specific Health Checkups and Specific Health Guidance standard [3]) as recorded in the NDB aggregate source files and the percentage with HbA1c ≥6.5%. The waist circumference criterion was interpreted as a central-adiposity-related surveillance indicator rather than a direct measure of visceral fat. Secondary and exploratory indicators reported in the tables included BMI ≥25 kg/m^2^, BMI ≥30 kg/m^2^, and BMI <18.5 kg/m^2^.

### Data completeness and handling of suppressed/unavailable cells

Cells displayed as “-” in the public NDB Open Data were treated as suppressed/unavailable cells. According to the official Ministry of Health, Labour and Welfare explanatory materials for the 10th NDB Open Data [6], cells are displayed as “-” when the aggregation unit is below the minimum reporting threshold, in principle fewer than 10. In addition, when only one item would otherwise be displayed as “-”, a cell with the smallest value of 10 or more may also be displayed as “-” to prevent back-calculation from totals. Therefore, cells displayed as “-” were not interpreted as true missing values and were not assumed to be zero counts. In the primary analysis, these cells were treated as unavailable public aggregate cells; their suppressed values were not recovered or imputed.

We assessed cell availability by indicator, sex, prefecture, and age band before interpreting the results. Because cell suppression/unavailability was uneven across indicators, we interpreted waist circumference analyses as the least affected by suppression/unavailability, HbA1c analyses as complete among men but qualified by suppression/unavailability among women, and BMI analyses as exploratory. To assess whether cell suppression was associated with stratum size, we calculated Pearson and Spearman correlations between the number of suppressed/unavailable age-band cells and the total publicly displayed waist-circumference examinee count across the seven age bands in each prefecture-sex stratum. Waist circumference counts were used because these data were complete across all prefectures, sexes, and age bands.

### Age adjustment and statistical analysis

For each sex and indicator, the denominator for a prefecture-sex-age-band combination was calculated by summing the publicly displayed counts across all result categories in the relevant NDB table. It therefore represented the number of examinees whose result for that indicator was included in the public table, not the total number of people who underwent a Specific Health Checkup in that prefecture-sex-age-band stratum. A combination was treated as available only when all category counts required to calculate the indicator-specific percentage were publicly displayed. Direct age adjustment was performed using the age-band distribution of the available indicator-specific displayed examinee counts pooled across prefectures as an internal standard. In the primary suppression-aware available-cell analysis, age-standardized percentages were calculated using only age bands in which the indicator-specific data were publicly available. The age weights were renormalized to sum to 1.0 across the available age bands within each prefecture-sex-indicator combination; that is, the age-standardized percentage was calculated as Σ(wa × pa)/Σwa over available age bands, where wa is the internal age-band weight and pa is the age-band-specific percentage. Age-band-specific percentages were truncated to four decimal places in the reshaped analytic dataset before age adjustment. This approach did not treat suppressed/unavailable cells as zero and did not estimate latent suppressed counts. It was used as a transparent descriptive calculation for public aggregate data with cell suppression/unavailability.

Complete-pairwise sensitivity analyses were conducted for selected correlations by restricting to prefectures with complete age-band cells for both indicators. We also assessed the robustness of ecological correlations using Spearman rank correlations, leave-one-out Pearson correlations, Cook’s distance and leverage diagnostics, and correlations excluding the prefecture with the largest Cook’s distance or leverage value. Illustrative stress tests were also conducted by assigning each suppressed count a value of 0 or 9 and recalculating the correlations while retaining the primary indicator-specific internal age weights. Because some “-” cells may reflect secondary masking rather than values strictly below 10, these substitution analyses were not interpreted as formal imputations or mathematical bounds.

Prefecture-level variation was summarized using the lowest and highest internally age-standardized percentages and their range in percentage points. Ecological correlations between selected indicators were assessed across prefectures and interpreted as descriptive co-patterning of aggregate surveillance indicators.

These ecological correlations were not interpreted as individual-level relationships, causal effects, or evidence of regional determinants. Because the public NDB tables represent administrative aggregate data for Specific Health Checkup examinees rather than a random sample designed for population inference, p-values and confidence intervals for the correlations were not presented. Statistical analyses were performed using IBM SPSS Statistics version 29 (IBM Corp., Armonk, NY, USA).

## Results

### Source data structure and completeness

The reshaped source data contained 672 age-band rows and 96 subtotal rows for each of BMI, HbA1c, and waist circumference. The age-band rows represented combinations of 48 geographic categories, consisting of 47 prefectures plus a category with unknown prefecture, two sexes, and seven age bands in the age-band files.

Table 1 summarizes cell availability for selected indicators. The pattern of suppression/unavailability was not uniform across indicators. Waist circumference criterion data were complete for all 47 prefectures, both sexes, and all age bands. HbA1c ≥6.5% was complete among men, but 17 of 47 prefectures among women had at least one suppressed/unavailable age-band cell. BMI ≥25 kg/m^2^ had suppressed/unavailable age-band cells in 36 of 47 prefectures among men and 15 of 47 prefectures among women.

**Table 1:** Cell availability of selected indicators.

**Indicator**	**Sex**	**Total prefectures**	**Complete prefectures, ** **n (%)**	**Total age-band cells**	**Complete age-band cells, ** **n (%)**	**Suppressed/unavailable age-** **band cells, n (%)**	**Interpretation**
Waist circumference criterion	Men	47	47 (100.0)	329	329 (100.0)	0 (0.0)	Complete
Waist circumference criterion	Women	47	47 (100.0)	329	329 (100.0)	0 (0.0)	Complete
HbA1c ≥6.5%	Men	47	47 (100.0)	329	329 (100.0)	0 (0.0)	Complete
HbA1c ≥6.5%	Women	47	30 (63.8)	329	293 (89.1)	36 (10.9)	Qualified by suppression/unavailability
BMI ≥25 kg/m^2^	Men	47	11 (23.4)	329	255 (77.5)	74 (22.5)	Exploratory
BMI ≥25 kg/m^2^	Women	47	32 (68.1)	329	298 (90.6)	31 (9.4)	Exploratory
BMI ≥30 kg/m^2^	Men	47	11 (23.4)	329	255 (77.5)	74 (22.5)	Exploratory
BMI ≥30 kg/m^2^	Women	47	32 (68.1)	329	298 (90.6)	31 (9.4)	Exploratory
BMI <18.5 kg/m^2^	Men	47	11 (23.4)	329	255 (77.5)	74 (22.5)	Exploratory
BMI <18.5 kg/m^2^	Women	47	32 (68.1)	329	298 (90.6)	31 (9.4)	Exploratory

HbA1c, hemoglobin A1c; BMI, body mass index. Waist criterion denotes meeting the waist circumference criterion. Total age-band cells were calculated as 47 prefectures × 7 age bands = 329 for each sex-indicator combination. Cells displayed as “-” in the public NDB Open Data were treated as suppressed/unavailable cells and were not assumed to be zero values. Percentages were calculated using 47 prefectures or 329 age-band cells as the denominator, as appropriate.

Supplementary Table S1 presents prefecture- and sex-specific internally age-standardized percentages of examinees meeting the waist circumference criterion, having HbA1c ≥6.5%, and having BMI ≥25 kg/m^2^, together with the corresponding complementary percentages and numbers of available age bands. It also reports the total publicly displayed waist-circumference examinee count across the seven age bands for each prefecture-sex stratum. Complementary percentages were calculated as 100 minus the corresponding internally age-standardized percentage using the same cell-availability and age-standardization rules and should therefore be interpreted together with the age-band availability information.

Suppressed/unavailable cells were more frequent in smaller prefecture-sex strata. For this analysis, stratum size was represented by the total publicly displayed waist-circumference examinee count across the seven age bands; waist circumference data were complete for all strata. Supplementary Table S2 presents both Pearson and Spearman correlations. The Spearman correlation between the number of suppressed/unavailable age-band cells and this stratum-size measure was −0.688 for BMI among men, −0.595 for BMI among women, and −0.704 for HbA1c among women.

### Prefecture-level variation

**Fig. 1:**
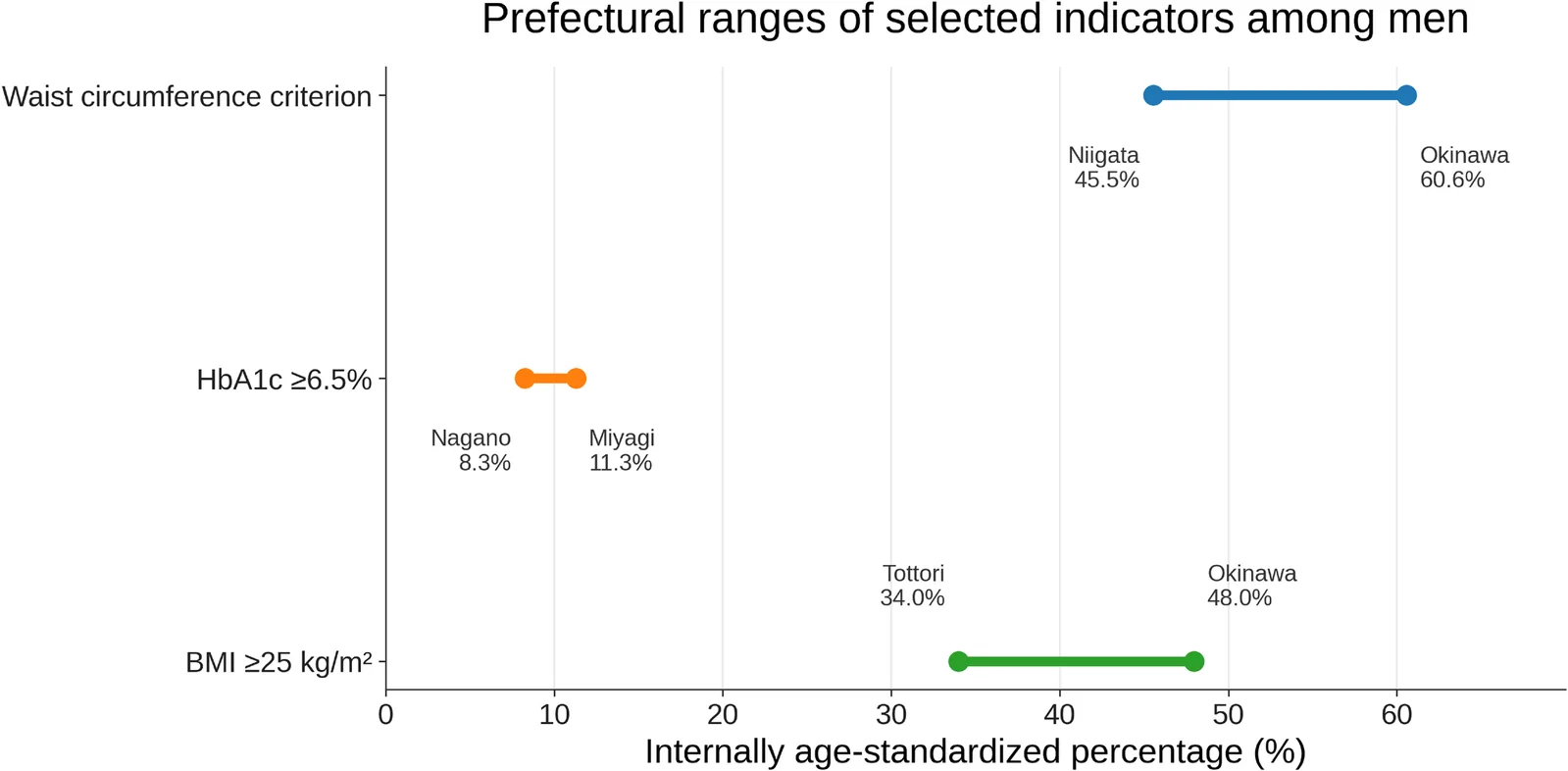
Prefectural ranges of selected indicators among men. Internally age-standardized percentages for selected indicators among men. Endpoints of each bar indicate the lowest and highest prefectures: waist circumference criterion, Niigata (45.5%) to Okinawa (60.6%); HbA1c ≥6.5%, Nagano (8.3%) to Miyagi (11.3%); BMI ≥25 kg/m^2^, Tottori (34.0%) to Okinawa (48.0%). BMI estimates should be interpreted as exploratory because of suppressed/unavailable age-band cells.

**Fig. 2:**
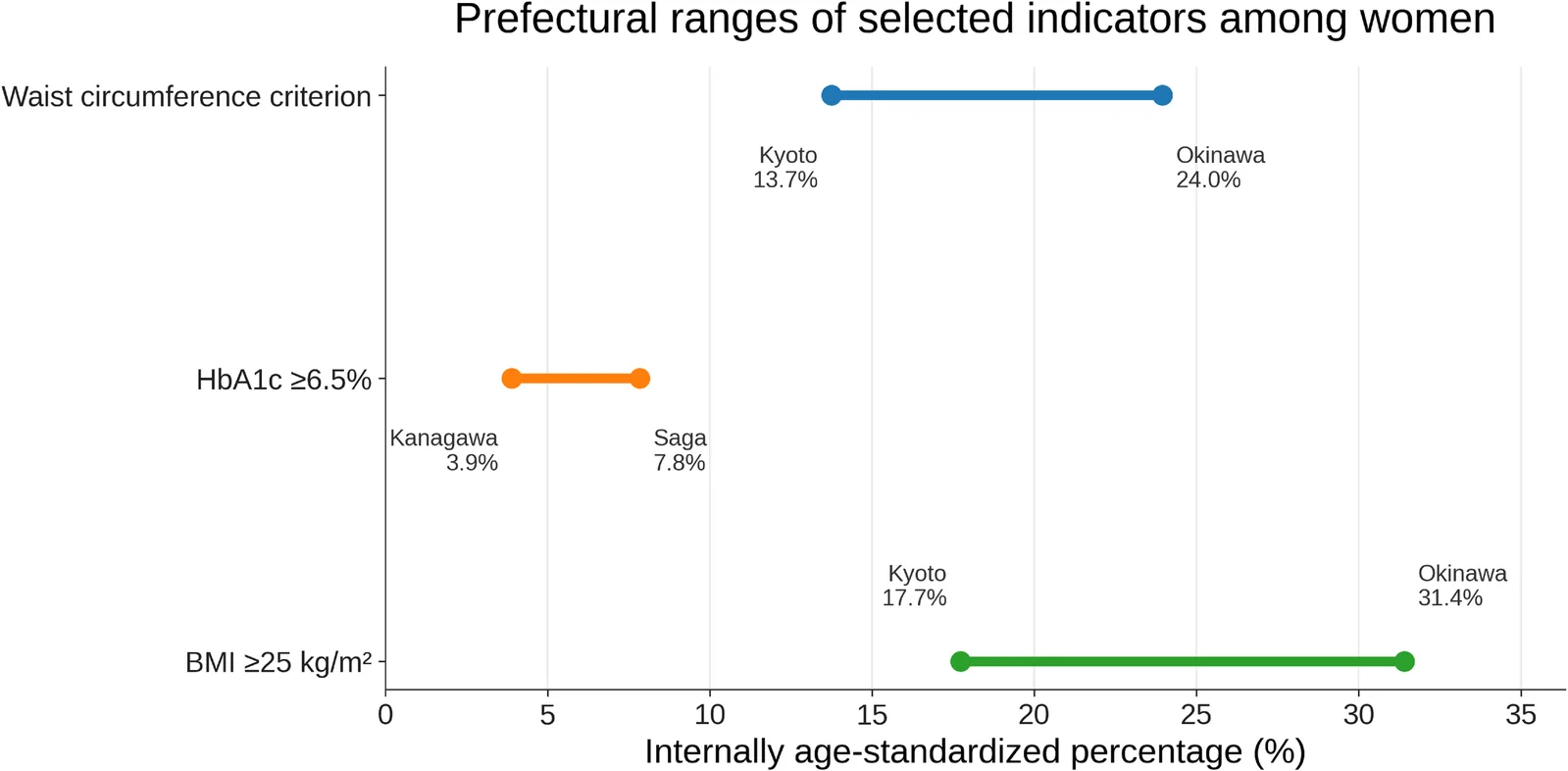
Prefectural ranges of selected indicators among women. Internally age-standardized percentages for selected indicators among women. Endpoints of each bar indicate the lowest and highest prefectures: waist circumference criterion, Kyoto (13.7%) to Okinawa (24.0%); HbA1c ≥6.5%, Kanagawa (3.9%) to Saga (7.8%); BMI ≥25 kg/m^2^, Kyoto (17.7%) to Okinawa (31.4%). HbA1c and BMI estimates for some prefectures were affected by suppressed/unavailable age-band cells; BMI estimates are exploratory.

The percentage meeting the waist circumference criterion showed marked prefecture-level variation (Table 2). Among men, the internally age-standardized percentage ranged from 45.5% in Niigata to 60.6% in Okinawa. Among women, it ranged from 13.7% in Kyoto to 24.0% in Okinawa. Okinawa had the highest percentage in both sexes. Based on the unrounded estimates, the corresponding ranges were 15.0 percentage points among men and 10.2 percentage points among women. Figures 1 and 2 visualize the ranges of selected indicators by sex.

**Table 2:** Internally age-standardized prefecture-level distribution.

**Indicator**	**Sex**	**Any age-band available**	**Complete age-bands**	**Lowest**	**Highest**	**Range, percentage points**
Waist circumference criterion	Men	47/47	47/47	Niigata, 45.5%	Okinawa, 60.6%	15.0
Waist circumference criterion	Women	47/47	47/47	Kyoto, 13.7%	Okinawa, 24.0%	10.2
HbA1c ≥6.5%	Men	47/47	47/47	Nagano, 8.3%	Miyagi, 11.3%	3.0
HbA1c ≥6.5%	Women	47/47	30/47	Kanagawa, 3.9%	Saga, 7.8%	3.9
BMI ≥25 kg/m^2^	Men	47/47	11/47	Tottori, 34.0%	Okinawa, 48.0%	14.0
BMI ≥25 kg/m^2^	Women	47/47	32/47	Kyoto, 17.7%	Okinawa, 31.4%	13.7
BMI ≥30 kg/m^2^	Men	47/47	11/47	Kyoto, 6.5%	Okinawa, 11.7%	5.1
BMI ≥30 kg/m^2^	Women	47/47	32/47	Hyogo, 4.1%	Okinawa, 8.5%	4.4
BMI <18.5 kg/m^2^	Men	47/47	11/47	Okinawa, 1.7%	Niigata, 3.6%	1.9
BMI <18.5 kg/m^2^	Women	47/47	32/47	Okinawa, 7.6%	Kyoto, 15.0%	7.4

Waist criterion denotes meeting the waist circumference criterion. Any age-band available indicates prefectures with at least one available age-band cell for the indicator; complete age-bands indicate prefectures with all seven age-band cells available. BMI estimates are exploratory because of substantial suppressed/unavailable age-band cells. Ranges were calculated from unrounded estimates; displayed endpoints are rounded to one decimal place.

HbA1c ≥6.5% showed a smaller but still observable prefecture-level range. Among men, the percentage ranged from 8.3% in Nagano to 11.3% in Miyagi. Among women, the available-cell estimate ranged from 3.9% in Kanagawa to 7.8% in Saga. BMI ≥25 kg/m^2^ estimates ranged from 34.0% in Tottori to 48.0% in Okinawa among men and from 17.7% in Kyoto to 31.4% in Okinawa among women; these BMI results are reported only as exploratory estimates because complete age-band data were available for 11 of 47 prefectures among men and 32 of 47 prefectures among women, reflecting substantial suppression/unavailability in BMI age-band cells. Therefore, BMI-based prefecture rankings should not be interpreted with the same confidence as waist circumference rankings.

### Ecological correlations and robustness checks

Table 3 shows prefecture-level ecological correlations and sensitivity checks. In available-cell analyses, the percentage meeting the waist circumference criterion was moderately correlated with the percentage with HbA1c ≥6.5% in both sexes (men, Pearson r = 0.553; women, r = 0.503). The corresponding Spearman correlations were 0.557 among men and 0.550 among women. In complete-pairwise analyses, the waist-HbA1c correlations were unchanged among men (47 prefectures) and stronger among women (Pearson r = 0.791; Spearman ρ = 0.806; n = 30), reflecting differences in cell availability and the prefectures included in the analysis.

**Table 3:** Prefecture-level ecological correlations and sensitivity checks.

**Sex**	**Pair**	**Analysis**	**N**	**Pearson r**	**Spearman ρ**	**Leave-one-out Pearson r range**	**Prefecture with largest Cook’s distance**
Men	Waist criterion vs HbA1c ≥6.5%	Available-cell	47	0.553	0.557	0.521–0.580	Okinawa
Men	Waist criterion vs HbA1c ≥6.5%	Complete-pairwise	47	0.553	0.557	0.521–0.580	Okinawa
Men	BMI ≥25 kg/m^2^ vs HbA1c ≥6.5%	Available-cell	47	0.468	0.452	0.435–0.517	Ishikawa
Men	BMI ≥25 kg/m^2^ vs HbA1c ≥6.5%	Complete-pairwise	11	0.586	0.573	0.561–0.626	Okinawa
Women	Waist criterion vs HbA1c ≥6.5%	Available-cell	47	0.503	0.550	0.472–0.570	Okinawa
Women	Waist criterion vs HbA1c ≥6.5%	Complete-pairwise	30	0.791	0.806	0.777–0.814	Okinawa
Women	BMI ≥25 kg/m^2^ vs HbA1c ≥6.5%	Available-cell	47	0.491	0.600	0.462–0.564	Tottori
Women	BMI ≥25 kg/m^2^ vs HbA1c ≥6.5%	Complete-pairwise	25	0.811	0.835	0.776–0.830	Okinawa

Correlations are ecological prefecture-level associations, not individual-level relationships or causal effects. Available-cell analyses used renormalized age weights over available age bands; complete-pairwise analyses restricted to prefectures with complete age-band cells for both indicators. Cells displayed as “-” were treated as suppressed/unavailable and were not assumed to be zero. Leave-one-out analyses, Cook’s distance and leverage diagnostics, and corresponding exclusion analyses assessed sensitivity to individual prefectures.

BMI ≥25 kg/m^2^ was also positively correlated with HbA1c ≥6.5%, but these correlations should be interpreted as exploratory because of BMI cell suppression/unavailability. Available-cell Pearson correlations were 0.468 among men and 0.491 among women, with Spearman correlations of 0.452 and 0.600, respectively. Complete-pairwise BMI correlations were calculated in smaller selected sets of prefectures and should not be interpreted as stronger evidence because the analytic prefectures were selected on the basis of complete availability of age-band cells.

Leave-one-out analyses showed that the positive direction of the reported correlations was not dependent on a single prefecture. For the waist-HbA1c association, leave-one-out Pearson correlations ranged from 0.521 to 0.580 among men, from 0.472 to 0.570 among women in the available-cell analysis, and from 0.777 to 0.814 among women in the complete-pairwise analysis. The prefecture with the largest Cook’s distance for the waist-HbA1c association was Okinawa in both sexes; excluding it yielded Pearson r = 0.541 among men, r = 0.522 among women in the available-cell analysis, and r = 0.785 among women in the complete-pairwise analysis (Supplementary Table S3).

In illustrative suppressed-count substitution analyses, assigning each suppressed count a value of 0 or 9 preserved positive waist-HbA1c and BMI-HbA1c correlations. Correlations among men changed minimally. Among women, the waist-HbA1c correlation increased to approximately 0.71 and the BMI-HbA1c correlation increased to approximately 0.63–0.64 under these substitution scenarios. These results supported a positive direction for the female ecological associations, but their magnitude was sensitive to how suppressed/unavailable cells were handled (Supplementary Table S4).

**Fig. 3:**
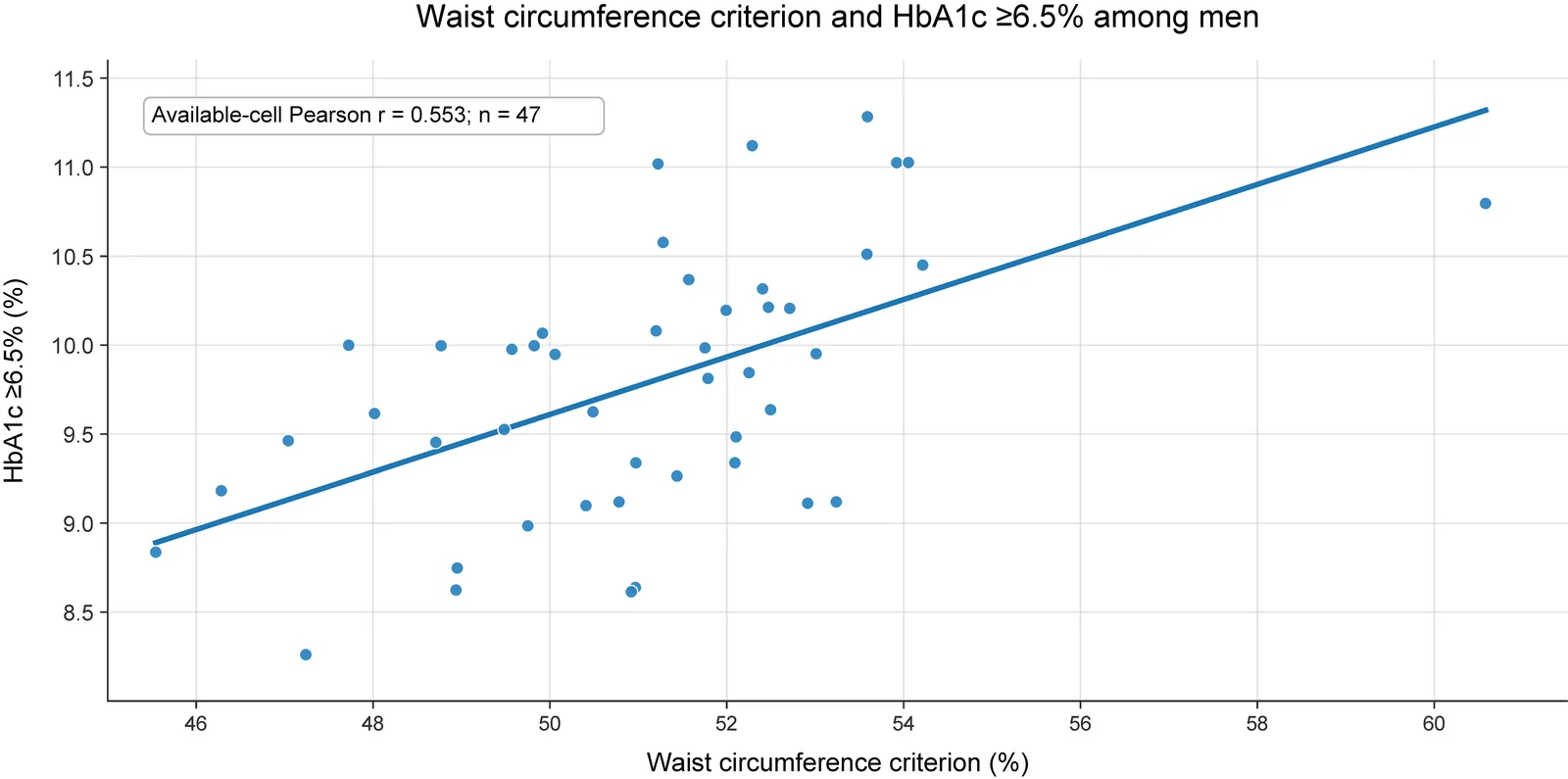
Waist circumference criterion and HbA1c ≥6.5% among men. Prefecture-level association between the internally age-standardized percentage meeting the waist circumference criterion and the percentage with HbA1c ≥6.5% among men.

**Fig. 4:**
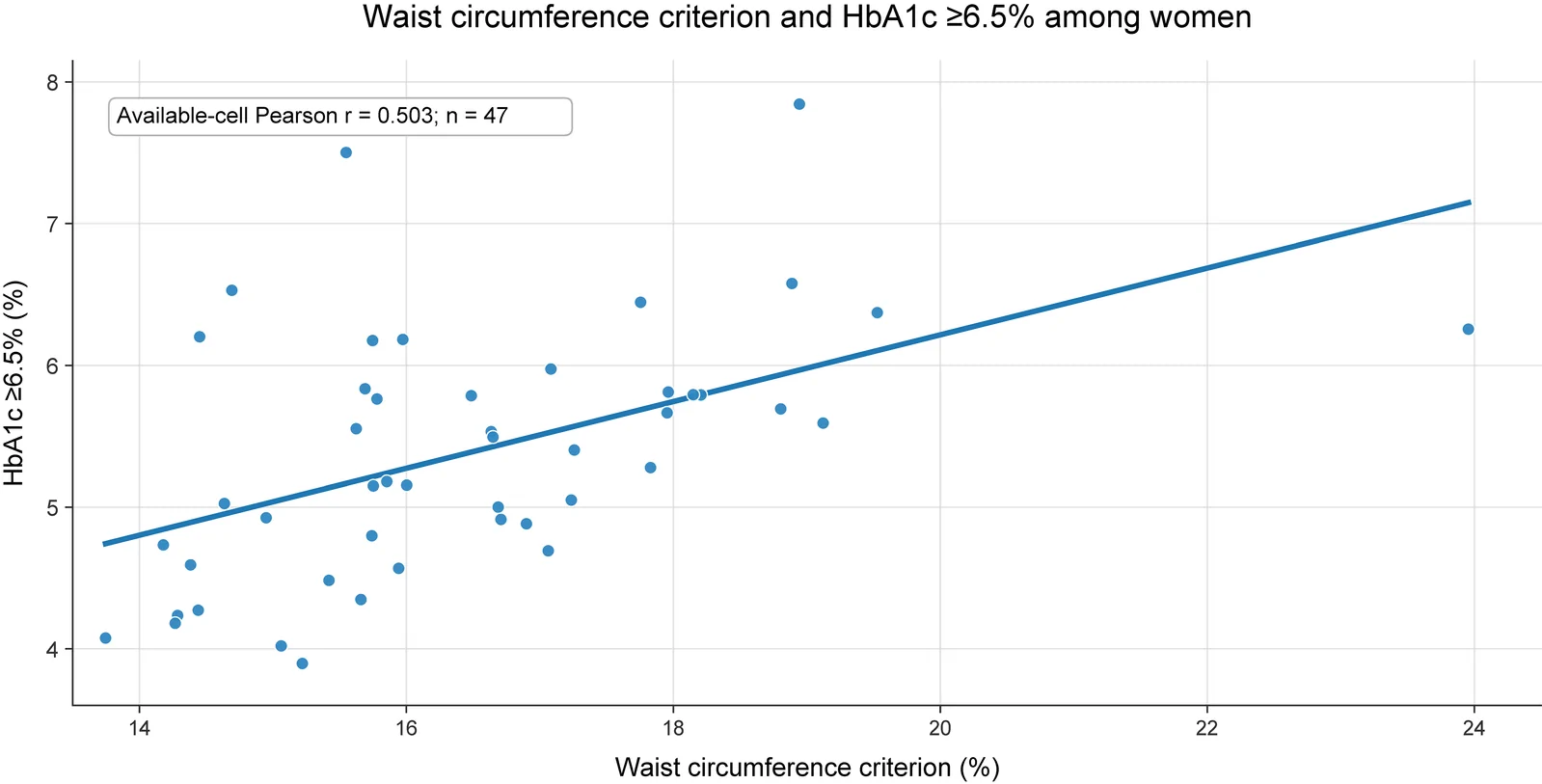
Waist circumference criterion and HbA1c ≥6.5% among women. Prefecture-level association between the internally age-standardized percentage meeting the waist circumference criterion and the percentage with HbA1c ≥6.5% among women. The HbA1c estimate used available-cell age adjustment when age-band cells were suppressed/unavailable.

Figures 3 and 4 show sex-specific scatter plots relating the prefecture-level percentage meeting the waist circumference criterion to the percentage with HbA1c ≥6.5%.

## Discussion

In this ecological analysis of the 10th NDB Open Data, we evaluated the usefulness and limitations of selected public aggregate indicators for prefecture-level cardiometabolic surveillance among Specific Health Checkup examinees aged 40–74 years. The main finding was not that prefecture-level differences identify etiologic determinants of cardiometabolic risk, but that the interpretability of public aggregate indicators differed substantially by data completeness and cell suppression/unavailability. Among the indicators examined, data for the percentage meeting the waist circumference criterion were complete across prefectures, sexes, and age bands and showed substantial prefecture-level variation. In contrast, HbA1c ≥6.5% among women and BMI-based indicators required suppression-aware interpretation.

The clearest finding concerns the waist circumference criterion. This indicator was complete across all prefectures, sexes, and age bands, and it showed substantial prefecture-level variation. In the Japanese Specific Health Checkup and Specific Health Guidance framework, the waist circumference criterion has a programmatic role in identifying central-adiposity-related metabolic risk and guiding preventive intervention. Therefore, the waist circumference criterion is relevant for descriptive regional surveillance using public aggregate checkup data. This interpretation is also biologically plausible because waist circumference is a central-adiposity-related measure [3]. However, waist circumference does not directly distinguish visceral fat from subcutaneous fat, muscle mass, or body frame. This limitation is particularly relevant for sex-specific interpretation because the same waist circumference may reflect different proportions of visceral and subcutaneous fat in men and women. The present aggregate analysis should therefore describe waist circumference as a practical surveillance proxy for central adiposity, not as a direct measurement of visceral adipose tissue.

The comparison between waist circumference and BMI further supports a cautious interpretation. BMI ≥25 kg/m^2^ was positively correlated with HbA1c ≥6.5%, but the available-cell correlations were slightly weaker than those for the waist circumference criterion. This pattern is compatible with the concept that central adiposity is metabolically relevant to insulin resistance and dysglycemia. Nevertheless, because BMI indicators had substantial cell suppression/unavailability and because the analysis was ecological, the present data should not be used to claim that waist circumference is superior to BMI at the individual level. Rather, in this NDB Open Data setting, waist circumference was both more complete and more interpretable for regional surveillance than BMI-based categories.

HbA1c ≥6.5% should be interpreted as an aggregate marker of glycemic burden rather than as a direct measure of newly detected diabetes. This indicator may include individuals with previously undiagnosed diabetes, individuals with diagnosed but untreated diabetes, and treated individuals with insufficient glycemic control. Because information on diabetes diagnosis, medication use, treatment duration, adherence, and clinical management was not available in the NDB Open Data, prefecture-level differences in HbA1c ≥6.5% may reflect both disease burden and differences in detection, treatment, and control.

The ecological correlations should be interpreted as descriptive co-patterning of aggregate surveillance indicators. Prefectures with higher percentages meeting the waist circumference criterion tended to have higher percentages with HbA1c ≥6.5%, but this prefecture-level association should not be interpreted as an individual-level relationship or causal effect. Differences in checkup participation, socioeconomic context, health behaviors, treatment patterns, and health care access may also contribute to geographic variation independently of adiposity. This interpretation is strongest for men, for whom HbA1c age-band cells were complete; among women, the direction was positive but the magnitude depended on cell availability and the prefectures included in the analysis. The female waist-HbA1c correlation should therefore be interpreted as directionally positive but not numerically stable. The observed prefecture-level patterns should be viewed as hypothesis-generating signals for regional surveillance rather than as evidence identifying specific determinants.

This work also illustrates the value and limitations of NDB Open Data for preventive medicine. The public tables enable transparent national-scale analyses without access to individual records, but the pre-aggregated structure limits adjustment for individual-level covariates and requires careful handling of cell suppression/unavailability. Previous reviews have shown the expanding use of the NDB for clinical epidemiology and health services research, while emphasizing limitations in clinical detail and validation [5]. Accordingly, the practical value of this analysis lies in identifying which indicators in the 10th NDB Open Data appear suitable for regional surveillance and which require caution because of suppression/unavailability. Waist circumference data may be suitable for transparent prefecture-level monitoring, whereas BMI-based rankings and female HbA1c correlations require cautious interpretation.

Prior large-scale analyses of Specific Health Checkup data have demonstrated the value of such data for characterizing metabolic syndrome-related markers, although those analyses addressed different questions and data structures [9]. Future studies linking the NDB Open Data with prefecture-level information on participation rates, treatment patterns, health behaviors, socioeconomic indicators, and medical care access could help clarify why waist circumference, BMI, and HbA1c patterns only partially overlap.

### Strengths and limitations

The strengths of this study include the use of nationwide public aggregate data, uniform stratification by prefecture, sex, and age band, and simultaneous evaluation of multiple cardiometabolic indicators. Because the source data are public and the analytic procedures are described explicitly, the findings can be independently checked using the original NDB Open Data source files.

Several limitations should be emphasized. First, this was an ecological analysis of aggregate data and cannot support individual-level inference. Second, the study population consisted of Specific Health Checkup examinees and should not be interpreted as the full Japanese adult population. Third, no external data were collected, so prefecture-level socioeconomic factors, health behaviors, medical treatment patterns, and checkup participation rates were not modeled. Fourth, BMI and female HbA1c analyses were affected by suppressed/unavailable age-band cells; available-cell estimates may be influenced by the age bands that remained publicly available, and complete-pairwise estimates use fewer prefectures selected on the basis of complete age-band availability. Fifth, internal age standardization supports comparison within the NDB checkup population but does not estimate age-standardized prevalence in the general population. Sixth, waist circumference was analyzed as a central-adiposity-related surveillance proxy, but it cannot directly separate visceral fat from subcutaneous fat or other components of body size. Seventh, although we added Spearman correlations, leave-one-out analyses, Cook’s distance and leverage diagnostics, and corresponding exclusion analyses, these procedures assess only the robustness of descriptive ecological correlations. They do not resolve ecological fallacy, unmeasured prefecture-level confounding, or uncertainty introduced by cell suppression/unavailability.

## Conclusions

The 10th NDB Open Data can support transparent descriptive surveillance of prefecture-level cardiometabolic indicators among Specific Health Checkup examinees in Japan, but the interpretability of each indicator depends on cell availability and suppression. Among the indicators examined, the percentage meeting the waist circumference criterion was the most complete and robust descriptive indicator. The percentage meeting the waist circumference criterion and the percentage with HbA1c ≥6.5% showed positive but incomplete geographic overlap; however, this ecological association should not be interpreted as an individual-level relationship or evidence of regional determinants. HbA1c analyses among women and BMI-based analyses require suppression-aware interpretation because of suppressed/unavailable age-band cells. These findings support the use of waist circumference criterion data for descriptive regional surveillance while emphasizing the need for transparent reporting of cell suppression and sensitivity analyses when using public aggregate NDB data.

## Abbreviations

BMI: body mass index
HbA1c: hemoglobin A1c
NDB: National Database of Health Insurance Claims and Specific Health Checkups of Japan
NCGG: National Center for Geriatrics and Gerontology
JSPS: Japan Society for the Promotion of Science
KAKENHI: Grants-in-Aid for Scientific Research

## Supplementary information

**Additional file 1:** **Supplementary Table S1.** Prefecture- and sex-specific internally age-standardized percentages of examinees meeting the waist circumference criterion, having HbA1c ≥6.5%, and having BMI ≥25 kg/m^2^, with corresponding complementary percentages and age-band availability. **Supplementary Table S2.** Numbers of suppressed/unavailable age-band cells and their correlations with displayed waist-circumference examinee counts, by indicator and sex. **Supplementary Table S3.** Exclusion analyses based on Cook’s distance and leverage for prefecture-level ecological correlations. **Supplementary Table S4.** Illustrative sensitivity analyses of Pearson correlations after assigning a value of 0 or 9 to each suppressed count.

## Declarations

### Availability of data and materials

The source data analyzed in this study are publicly available as the 10th NDB Open Data from the Ministry of Health, Labour and Welfare, Japan [6]. Additional analytic documentation is available from the corresponding author upon reasonable request.

## References

[1] Kohro T, Furui Y, Mitsutake N, Fujii R, Morita H, Oku S, . The Japanese national health screening and intervention program aimed at preventing worsening of the metabolic syndrome. Int Heart J. 2008;49:193–203. doi: 10.1536/ihj.49.193. 18475019

[2] Tsushita K, Hosler AS, Miura K, Ito Y, Fukuda T, Kitamura A, . Rationale and descriptive analysis of Specific Health Guidance: the nationwide lifestyle intervention program targeting metabolic syndrome in Japan. J Atheroscler Thromb. 2018;25:308–22. doi: 10.5551/jat.42010. 29238010 PMC5906184

[3] Ministry of Health, Labour and Welfare, Japan. Specific Health Checkups and Specific Health Guidance. https://www.mhlw.go.jp/content/12401000/001328080.pdf. Accessed 29 May 2026. Japanese.

[4] Yasunaga H. Updated information on NDB. Ann Clin Epidemiol. 2024;6:73–6. doi: 10.37737/ace.24011. 39034945 PMC11254583

[5] Hirose N, Ishimaru M, Morita K, Yasunaga H. A review of studies using the Japanese National Database of Health Insurance Claims and Specific Health Checkups. Ann Clin Epidemiol. 2020;2:13–26. doi: 10.37737/ace.2.1_13.

[6] Ministry of Health, Labour and Welfare, Japan. The 10th NDB Open Data and explanatory materials. https://www.mhlw.go.jp/stf/seisakunitsuite/bunya/0000177221_00016.html. Accessed 25 May 2026. Japanese.

[7] Morgenstern H. Ecologic studies in epidemiology: concepts, principles, and methods. Annu Rev Public Health. 1995;16:61–81. doi: 10.1146/annurev.pu.16.050195.000425. 7639884

[8] Robinson WS. Ecological correlations and the behavior of individuals. Am Sociol Rev. 1950;15:351–7. doi: 10.2307/2087176. 19179346

[9] Seto H, Toki H, Kitora S, Oyama A, Yamamoto R. Seasonal variations of the prevalence of metabolic syndrome and its markers using big-data of health check-ups. Environ Health Prev Med. 2024;29:2. doi: 10.1265/ehpm.23-00216. 38246652 PMC10808004

