# Supplementary material for: Geographic variation in proportions meeting the waist circumference criterion and having elevated hemoglobin A1c among Specific Health Checkup examinees in Japan: an ecological analysis of the 10th NDB Open Data
Source: Environ Health Prev Med. 2026 Aug 18;31:54. doi: 10.1265/ehpm.26-00125 (PMC13500941; doi:10.1265/ehpm.26-00125)
Supplement: Supplementary file 1 [file ehpm-31-054-s001.pdf]

Supplementary Material

Supplementary Tables S1–S4

Geographic variation in proportions meeting the waist circumference criterion and having elevated hemoglobin A1c among Specific Health Checkup examinees in Japan: an ecological analysis of the 10th NDB Open Data

Hirotaka Nakashima, Takahiro Kamihara, and Takuya Omura

Supplementary Table S1. Prefecture- and sex-specific internally age-standardized percentages of examinees meeting the waist circumference criterion, having HbA1c ≥6.5%, and having BMI ≥25 kg/m², with corresponding complementary percentages and age-band availability.

Panel A. Men

Prefecture	Waist circumference criterion			HbA1c ≥6.5%			BMI ≥25 kg/m²			Displayed waist-circumference examinees, n
	Meeting, %	Complement, %	Available age bands, n	Meeting, %	Complement, %	Available age bands, n	Meeting, %	Complement, %	Available age bands, n	
Hokkaido	52.471	47.529	7	10.213	89.787	7	41.583	58.417	7	542,422
Aomori	51.570	48.430	7	10.368	89.632	7	42.156	57.844	5	154,682
Iwate	50.408	49.592	7	9.099	90.901	7	41.046	58.954	5	164,281
Miyagi	53.591	46.409	7	11.283	88.717	7	40.145	59.855	7	315,039
Akita	53.238	46.762	7	9.120	90.880	7	41.022	58.978	5	122,367
Yamagata	47.042	52.958	7	9.463	90.537	7	38.676	61.324	5	160,371
Fukushima	52.712	47.288	7	10.207	89.793	7	40.953	59.047	5	243,196
Ibaraki	52.288	47.712	7	11.120	88.880	7	40.198	59.802	5	370,656
Tochigi	51.992	48.008	7	10.196	89.804	7	39.778	60.222	5	263,809
Gunma	51.201	48.799	7	10.080	89.920	7	38.107	61.893	5	252,858
Saitama	51.788	48.212	7	9.813	90.187	7	37.848	62.152	7	950,522
Chiba	53.010	46.990	7	9.951	90.049	7	38.840	61.160	7	789,327
Tokyo	50.967	49.033	7	8.638	91.362	7	35.886	64.114	7	1,856,199
Kanagawa	50.921	49.079	7	8.614	91.386	7	36.412	63.588	7	1,200,638
Niigata	45.543	54.457	7	8.837	91.163	7	34.103	65.897	5	316,623
Toyama	51.280	48.720	7	10.577	89.423	7	35.606	64.394	5	148,369
Ishikawa	51.223	48.777	7	11.018	88.982	7	35.800	64.200	5	150,178
Fukui	52.252	47.748	7	9.845	90.155	7	36.098	63.902	5	97,673
Yamanashi	48.770	51.230	7	9.997	90.003	7	36.672	63.328	5	112,775
Nagano	47.240	52.760	7	8.260	91.740	7	34.257	65.743	5	284,966
Gifu	46.284	53.716	7	9.183	90.817	7	35.406	64.594	5	261,469
Shizuoka	47.725	52.275	7	10.000	90.000	7	35.125	64.875	7	496,420
Aichi	49.570	50.430	7	9.977	90.023	7	36.056	63.944	7	992,040
Mie	49.916	50.084	7	10.067	89.933	7	37.097	62.903	5	243,853

Supplementary Table S1 (continued)

Panel A. Men (continued)

Prefecture	Waist circumference criterion			HbA1c ≥6.5%			BMI ≥25 kg/m²			Displayed waist-circumference examinees, n
	Meeting, %	Complement, %	Available age bands, n	Meeting, %	Complement, %	Available age bands, n	Meeting, %	Complement, %	Available age bands, n	
Shiga	49.750	50.250	7	8.985	91.015	7	35.457	64.543	5	184,831
Kyoto	48.952	51.048	7	8.748	91.252	7	34.106	65.894	5	293,880
Osaka	52.105	47.895	7	9.484	90.516	7	36.559	63.441	7	969,081
Hyogo	50.782	49.218	7	9.119	90.881	7	35.949	64.051	5	654,979
Nara	51.437	48.563	7	9.265	90.735	7	37.144	62.856	5	147,895
Wakayama	54.216	45.784	7	10.450	89.550	7	37.968	62.032	5	104,092
Tottori	48.938	51.062	7	8.624	91.376	7	33.980	66.020	4	64,273
Shimane	48.017	51.983	7	9.615	90.385	7	34.639	65.361	4	89,282
Okayama	50.058	49.942	7	9.948	90.052	7	36.784	63.216	5	226,480
Hiroshima	50.487	49.513	7	9.625	90.375	7	36.553	63.447	5	340,004
Yamaguchi	48.710	51.290	7	9.453	90.547	7	35.837	64.163	5	156,988
Tokushima	52.092	47.908	7	9.338	90.662	7	40.253	59.747	5	84,639
Kagawa	50.972	49.028	7	9.338	90.662	7	38.539	61.461	5	118,815
Ehime	49.483	50.517	7	9.526	90.474	7	39.790	60.210	5	159,267
Kochi	51.754	48.246	7	9.984	90.016	7	38.988	61.012	5	81,345
Fukuoka	52.405	47.595	7	10.316	89.684	7	36.940	63.060	7	583,079
Saga	53.921	46.079	7	11.025	88.975	7	37.717	62.283	5	96,864
Nagasaki	52.915	47.085	7	9.112	90.888	7	39.239	60.761	5	149,423
Kumamoto	54.055	45.945	7	11.026	88.974	7	40.266	59.734	5	204,254
Oita	49.822	50.178	7	9.997	90.003	7	40.414	59.586	5	138,806
Miyazaki	52.496	47.504	7	9.637	90.363	7	39.557	60.443	5	126,488
Kagoshima	53.585	46.415	7	10.511	89.489	7	40.980	59.020	5	187,392
Okinawa	60.581	39.419	7	10.795	89.205	7	47.975	52.025	7	155,669

Supplementary Table S1 (continued)

Panel B. Women

Prefecture	Waist circumference criterion			HbA1c ≥6.5%			BMI ≥25 kg/m²			Displayed waist-circumference examinees, n
	Meeting, %	Complement, %	Available age bands, n	Meeting, %	Complement, %	Available age bands, n	Meeting, %	Complement, %	Available age bands, n	
Hokkaido	16.688	83.312	7	5.001	94.999	7	24.544	75.456	7	432,773
Aomori	17.829	82.171	7	5.278	94.722	7	26.921	73.079	7	138,409
Iwate	17.953	82.047	7	5.665	94.335	5	26.411	73.589	7	144,757
Miyagi	18.207	81.793	7	5.792	94.208	7	24.629	75.371	7	271,856
Akita	18.149	81.851	7	5.793	94.207	5	25.855	74.145	7	109,684
Yamagata	14.692	85.308	7	6.530	93.470	5	24.618	75.382	5	145,521
Fukushima	18.888	81.112	7	6.579	93.421	5	26.507	73.493	7	212,493
Ibaraki	17.083	82.917	7	5.975	94.025	7	24.204	75.796	7	298,760
Tochigi	17.258	82.742	7	5.403	94.597	7	24.640	75.360	7	208,743
Gunma	16.635	83.365	7	5.532	94.468	7	22.968	77.032	7	210,224
Saitama	16.708	83.292	7	4.914	95.086	7	22.209	77.791	7	770,146
Chiba	17.063	82.937	7	4.693	95.307	7	22.165	77.835	7	656,436
Tokyo	15.062	84.938	7	4.021	95.979	7	19.170	80.830	7	1,622,582
Kanagawa	15.220	84.780	7	3.897	96.103	7	19.605	80.395	7	939,019
Niigata	14.383	85.617	7	4.593	95.407	7	20.525	79.475	7	270,316
Toyama	17.236	82.764	7	5.051	94.949	7	19.698	80.302	5	127,917
Ishikawa	16.649	83.351	7	5.494	94.506	7	19.602	80.398	5	135,045
Fukui	15.973	84.027	7	6.183	93.817	5	20.111	79.889	5	84,611
Yamanashi	14.451	85.549	7	6.202	93.798	5	20.722	79.278	5	99,466
Nagano	14.286	85.714	7	4.236	95.764	7	19.756	80.244	7	242,617
Gifu	14.179	85.821	7	4.734	95.266	7	19.833	80.167	7	216,581
Shizuoka	14.949	85.051	7	4.926	95.074	7	20.519	79.481	7	403,702
Aichi	15.853	84.147	7	5.181	94.819	7	20.380	79.620	7	768,475
Mie	15.752	84.248	7	5.150	94.850	7	21.008	78.992	7	202,589

Supplementary Table S1 (continued)

Panel B. Women (continued)

Prefecture	Waist circumference criterion			HbA1c ≥6.5%			BMI ≥25 kg/m²			Displayed waist-circumference examinees, n
	Meeting, %	Complement, %	Available age bands, n	Meeting, %	Complement, %	Available age bands, n	Meeting, %	Complement, %	Available age bands, n	
Shiga	14.441	85.559	7	4.273	95.727	7	19.182	80.818	5	156,155
Kyoto	13.746	86.254	7	4.076	95.924	7	17.728	82.272	5	254,623
Osaka	15.659	84.341	7	4.348	95.652	7	19.954	80.046	7	815,532
Hyogo	14.268	85.732	7	4.181	95.819	7	18.732	81.268	7	549,296
Nara	14.637	85.363	7	5.026	94.974	5	19.298	80.702	7	135,501
Wakayama	15.747	84.253	7	6.176	93.824	5	20.245	79.755	5	91,859
Tottori	15.549	84.451	7	7.502	92.498	3	20.541	79.459	5	55,813
Shimane	15.690	84.310	7	5.835	94.165	5	19.813	80.187	4	78,820
Okayama	16.002	83.998	7	5.155	94.845	7	21.892	78.108	7	192,365
Hiroshima	15.420	84.580	7	4.484	95.516	7	20.463	79.537	7	267,239
Yamaguchi	15.741	84.259	7	4.799	95.201	7	20.285	79.715	5	130,561
Tokushima	15.780	84.220	7	5.763	94.237	5	23.446	76.554	5	78,734
Kagawa	15.942	84.058	7	4.569	95.431	7	22.459	77.541	7	105,304
Ehime	15.624	84.376	7	5.553	94.447	5	23.118	76.882	5	141,861
Kochi	17.755	82.245	7	6.446	93.554	5	24.095	75.905	7	76,091
Fukuoka	16.899	83.101	7	4.883	95.117	7	20.629	79.371	7	505,065
Saga	18.944	81.056	7	7.844	92.156	5	22.414	77.586	5	84,785
Nagasaki	17.962	82.038	7	5.812	94.188	5	23.682	76.318	7	138,571
Kumamoto	18.804	81.196	7	5.693	94.307	7	23.736	76.264	7	186,411
Oita	16.486	83.514	7	5.786	94.214	5	23.681	76.319	5	121,618
Miyazaki	19.528	80.472	7	6.373	93.627	5	24.711	75.289	7	112,792
Kagoshima	19.122	80.878	7	5.593	94.407	7	25.323	74.677	7	163,300
Okinawa	23.956	76.044	7	6.256	93.744	7	31.410	68.590	7	143,767

Notes: Complementary percentages were calculated as 100 minus the corresponding internally age-standardized percentage using the same cell-availability and age-standardization rules. Unrounded age-standardized values were used for correlation analyses. HbA1c estimates among women and BMI estimates used available-cell age adjustment when age-band cells were suppressed/unavailable. BMI estimates are exploratory because of suppressed/unavailable age-band cells. BMI, body mass index; HbA1c, hemoglobin A1c.

Supplementary Table S2. Numbers of suppressed/unavailable age-band cells and their correlations with displayed waist-circumference examinee counts, by indicator and sex.

Panel A1. Men: prefecture-specific counts

Prefecture	Sex	Displayed waist-circumference examinees, n	Suppressed/unavailable HbA1c age-band cells, n	Suppressed/unavailable BMI age-band cells, n
Hokkaido	Men	542,422	0	0
Aomori	Men	154,682	0	2
Iwate	Men	164,281	0	2
Miyagi	Men	315,039	0	0
Akita	Men	122,367	0	2
Yamagata	Men	160,371	0	2
Fukushima	Men	243,196	0	2
Ibaraki	Men	370,656	0	2
Tochigi	Men	263,809	0	2
Gunma	Men	252,858	0	2
Saitama	Men	950,522	0	0
Chiba	Men	789,327	0	0
Tokyo	Men	1,856,199	0	0
Kanagawa	Men	1,200,638	0	0
Niigata	Men	316,623	0	2
Toyama	Men	148,369	0	2
Ishikawa	Men	150,178	0	2
Fukui	Men	97,673	0	2
Yamanashi	Men	112,775	0	2
Nagano	Men	284,966	0	2
Gifu	Men	261,469	0	2
Shizuoka	Men	496,420	0	0
Aichi	Men	992,040	0	0
Mie	Men	243,853	0	2
Shiga	Men	184,831	0	2
Kyoto	Men	293,880	0	2

Supplementary Table S2 (continued)

Panel A1. Men: prefecture-specific counts (continued)

Prefecture	Sex	Displayed waist-circumference examinees, n	Suppressed/unavailable HbA1c age-band cells, n	Suppressed/unavailable BMI age-band cells, n
Osaka	Men	969,081	0	0
Hyogo	Men	654,979	0	2
Nara	Men	147,895	0	2
Wakayama	Men	104,092	0	2
Tottori	Men	64,273	0	3
Shimane	Men	89,282	0	3
Okayama	Men	226,480	0	2
Hiroshima	Men	340,004	0	2
Yamaguchi	Men	156,988	0	2
Tokushima	Men	84,639	0	2
Kagawa	Men	118,815	0	2
Ehime	Men	159,267	0	2
Kochi	Men	81,345	0	2
Fukuoka	Men	583,079	0	0
Saga	Men	96,864	0	2
Nagasaki	Men	149,423	0	2
Kumamoto	Men	204,254	0	2
Oita	Men	138,806	0	2
Miyazaki	Men	126,488	0	2
Kagoshima	Men	187,392	0	2
Okinawa	Men	155,669	0	0

Supplementary Table S2 (continued)

Panel A2. Women: prefecture-specific counts

Prefecture	Sex	Displayed waist-circumference examinees, n	Suppressed/unavailable HbA1c age-band cells, n	Suppressed/unavailable BMI age-band cells, n
Hokkaido	Women	432,773	0	0
Aomori	Women	138,409	0	0
Iwate	Women	144,757	2	0
Miyagi	Women	271,856	0	0
Akita	Women	109,684	2	0
Yamagata	Women	145,521	2	2
Fukushima	Women	212,493	2	0
Ibaraki	Women	298,760	0	0
Tochigi	Women	208,743	0	0
Gunma	Women	210,224	0	0
Saitama	Women	770,146	0	0
Chiba	Women	656,436	0	0
Tokyo	Women	1,622,582	0	0
Kanagawa	Women	939,019	0	0
Niigata	Women	270,316	0	0
Toyama	Women	127,917	0	2
Ishikawa	Women	135,045	0	2
Fukui	Women	84,611	2	2
Yamanashi	Women	99,466	2	2
Nagano	Women	242,617	0	0
Gifu	Women	216,581	0	0
Shizuoka	Women	403,702	0	0
Aichi	Women	768,475	0	0
Mie	Women	202,589	0	0
Shiga	Women	156,155	0	2
Kyoto	Women	254,623	0	2

Supplementary Table S2 (continued)

Panel A2. Women: prefecture-specific counts (continued)

Prefecture	Sex	Displayed waist-circumference examinees, n	Suppressed/unavailable HbA1c age-band cells, n	Suppressed/unavailable BMI age-band cells, n
Osaka	Women	815,532	0	0
Hyogo	Women	549,296	0	0
Nara	Women	135,501	2	0
Wakayama	Women	91,859	2	2
Tottori	Women	55,813	4	2
Shimane	Women	78,820	2	3
Okayama	Women	192,365	0	0
Hiroshima	Women	267,239	0	0
Yamaguchi	Women	130,561	0	2
Tokushima	Women	78,734	2	2
Kagawa	Women	105,304	0	0
Ehime	Women	141,861	2	2
Kochi	Women	76,091	2	0
Fukuoka	Women	505,065	0	0
Saga	Women	84,785	2	2
Nagasaki	Women	138,571	2	0
Kumamoto	Women	186,411	0	0
Oita	Women	121,618	2	2
Miyazaki	Women	112,792	2	0
Kagoshima	Women	163,300	0	0
Okinawa	Women	143,767	0	0

Supplementary Table S2 (continued)

Panel B. Correlations between suppressed/unavailable age-band cell counts and stratum size

Indicator	Sex	Prefectures, n	Pearson r	Spearman ρ
HbA1c ≥6.5%	Women	47	-0.431	-0.704
BMI ≥25 kg/m²	Men	47	-0.734	-0.688
BMI ≥25 kg/m²	Women	47	-0.383	-0.595

Notes: Stratum size was the total publicly displayed waist-circumference examinee count across the seven age bands. Correlations were not calculated for the waist circumference criterion or male HbA1c because every prefecture had zero suppressed/unavailable cells. Negative correlations indicate more frequent suppression/unavailability in smaller strata. BMI, body mass index; HbA1c, hemoglobin A1c.

Supplementary Table S3. Exclusion analyses based on Cook's distance and leverage for prefecture-level ecological correlations.

Panel A. Cook's distance-based exclusion analyses

Sex	Indicator pair	Analysis	N	Original Pearson r	Prefecture with largest Cook's D	Cook's D	Pearson r after exclusion
Men	Waist criterion vs HbA1c ≥6.5%	Available-cell	47	0.553	Okinawa	0.259	0.541
Men	Waist criterion vs HbA1c ≥6.5%	Complete-pairwise	47	0.553	Okinawa	0.259	0.541
Men	BMI ≥25 kg/m² vs HbA1c ≥6.5%	Available-cell	47	0.468	Ishikawa	0.098	0.517
Men	BMI ≥25 kg/m² vs HbA1c ≥6.5%	Complete-pairwise	11	0.586	Okinawa	1.309	0.564
Women	Waist criterion vs HbA1c ≥6.5%	Available-cell	47	0.503	Okinawa	0.634	0.522
Women	Waist criterion vs HbA1c ≥6.5%	Complete-pairwise	30	0.791	Okinawa	2.256	0.785
Women	BMI ≥25 kg/m² vs HbA1c ≥6.5%	Available-cell	47	0.491	Tottori	0.147	0.564
Women	BMI ≥25 kg/m² vs HbA1c ≥6.5%	Complete-pairwise	25	0.811	Okinawa	0.503	0.776

Panel B. Leverage-based exclusion analyses

Sex	Indicator pair	Analysis	N	Original Pearson r	Prefecture with largest leverage	Leverage	Pearson r after exclusion
Men	Waist criterion vs HbA1c ≥6.5%	Available-cell	47	0.553	Okinawa	0.329	0.541
Men	Waist criterion vs HbA1c ≥6.5%	Complete-pairwise	47	0.553	Okinawa	0.329	0.541
Men	BMI ≥25 kg/m² vs HbA1c ≥6.5%	Available-cell	47	0.468	Okinawa	0.315	0.435
Men	BMI ≥25 kg/m² vs HbA1c ≥6.5%	Complete-pairwise	11	0.586	Okinawa	0.747	0.564
Women	Waist criterion vs HbA1c ≥6.5%	Available-cell	47	0.503	Okinawa	0.370	0.522
Women	Waist criterion vs HbA1c ≥6.5%	Complete-pairwise	30	0.791	Okinawa	0.531	0.785
Women	BMI ≥25 kg/m² vs HbA1c ≥6.5%	Available-cell	47	0.491	Okinawa	0.266	0.487
Women	BMI ≥25 kg/m² vs HbA1c ≥6.5%	Complete-pairwise	25	0.811	Okinawa	0.451	0.776

Notes: HbA1c ≥6.5% was modeled as the dependent variable and the adiposity-related indicator as the predictor. Original Pearson r is the correlation before exclusion. For each diagnostic, the post-exclusion correlation removes only the prefecture with the largest corresponding value. Complete-pairwise analyses include only prefectures with all seven age-band cells available for both indicators. These analyses assess sensitivity to individual prefectures. BMI, body mass index; HbA1c, hemoglobin A1c; waist criterion, meeting the waist circumference criterion.

Supplementary Table S4. Illustrative sensitivity analyses of Pearson correlations after assigning a value of 0 or 9 to each suppressed count.

Sex	Indicator pair	Replacement assigned to each suppressed count	N	Original available-cell Pearson r	Pearson r after substitution
Men	Waist criterion vs HbA1c ≥6.5%	0	47	0.553	0.553
Men	Waist criterion vs HbA1c ≥6.5%	9	47	0.553	0.553
Men	BMI ≥25 kg/m² vs HbA1c ≥6.5%	0	47	0.468	0.473
Men	BMI ≥25 kg/m² vs HbA1c ≥6.5%	9	47	0.468	0.473
Women	Waist criterion vs HbA1c ≥6.5%	0	47	0.503	0.711
Women	Waist criterion vs HbA1c ≥6.5%	9	47	0.503	0.712
Women	BMI ≥25 kg/m² vs HbA1c ≥6.5%	0	47	0.491	0.634
Women	BMI ≥25 kg/m² vs HbA1c ≥6.5%	9	47	0.491	0.637

Notes: The primary indicator-specific internal age weights were retained. Because some “-” cells may reflect secondary masking rather than values strictly below 10, the substitutions are not formal imputations or mathematical bounds. Where no suppressed counts were present, the substituted and original correlations are identical. BMI, body mass index; HbA1c, hemoglobin A1c; waist criterion, meeting the waist circumference criterion.
